# The characteristics of online gerontophobia expressions in South Korea

**DOI:** 10.3389/fpsyg.2023.1290443

**Published:** 2023-12-15

**Authors:** Sohui Kim, Min Ho Ryu

**Affiliations:** Dong-A University, Busan, Republic of Korea

**Keywords:** gerontophobia, online hate, LDA topic modeling, deep learning, text analysis

## Abstract

Recently, South Korea has been transitioning into a super-aged society. The purpose of this paper is to identify the patterns and underlying causes of gerontophobia expressions in South Korea. This paper refines the patterns of gerontophobia expressions into five types: “Fear of Aging,” “Resource Burden,” “Social Isolation,” “Criticism of Social Behavior,” and “Stereotypes of Political Orientation.” Based on these types, this study develops a deep learning algorithm to detect the type of gerontophobia expressions. To do this, kc-BERT was used and 760,140 news comments (for six years from May 1, 2017, to June 31, 2021) in Naver news was used. The result shows that “Fear of Aging” type exhibited a significant decreasing trend, while the other types showed no meaningful changes. The results of topic modeling on news articles indicated that various aspects of elderly life, unresolved historical events, COVID−19, digital and financial exclusion, economic and social welfare, and other critical societal issues co-occur and contribute to gerontophobia. This study provides a framework to understand the characteristics of online gerontophobia, offering insights into its underlying causes, and providing practical implications for policy makers.

## 1 Introduction

Currently, South Korean society is experiencing both aging population and low birth rate phenomena simultaneously, leading to a rapid transformation in its demographic structure. As of 2022, the proportion of the population aged 65 and above stands at 17.5% of the total population. This ratio is projected to continue increasing, with estimates indicating that it will reach 20.6% by the year 2025, signifying the transition into a super-aged society (KOSIS_a, 2022). On the other hand, the total fertility rate in 2022 has been recorded at 0.78 (KOSIS_b, 2022), highlighting the acceleration of the low birth rate trend each year. Such circumstances are deemed uncommon on a global scale.

In Korean society, the rapid transformation of the demographic structure has led to an escalating intergenerational conflict. In South Korea’s case, rapid economic development has been accompanied by industrialization and urbanization. Through this process, each generation has experienced distinct environments and cultures, forming unique social structures characterized by differing value systems. As a result, traditional values were changed, leading to a diminished sense of authority among the elderly and their portrayal as non-productive. This phenomenon has given rise to the proliferation of negative biases (Chung et al., 2021), and it is escalating to the extent of gerontophobia.

In general, hatred refers to harboring animosity and aversion toward specific groups for various reasons such as gender, religion, race, and disabilities (Jay, 2009). This encompasses more than mere profanity or vulgar language; it can be defined as an act of disparaging specific groups based on prejudice (Kim and Jung, 2021). In today’s digital age, the advancement of online digital platforms has facilitated the rapid dissemination of such social perceptions, particularly through mediums like social media. The content exposed online, and the public’s perceptions have been shown to influence real-life attitudes, a fact supported by various studies (Fox et al., 2015; Kim M. et al., 2020; Elsaesser et al., 2021). Therefore, efforts to comprehend and address hate speech manifesting online are essential.

Considering the reality in Korea, conflicts between the elderly and other generations will likely escalate into societal disputes. This situation amplifies social unrest and ultimately incurs societal costs, making the resolution of intergenerational conflicts a serious imperative (Won and Han, 2019). Moreover, beyond Korea, reports indicate that trends in social media analysis are revealing a deepening of age discrimination and generational tensions on social media platforms in various other countries as well (Meisner, 2021).

From this perspective, gerontophobia should be recognized as a global issue, not limited to Korea. Nevertheless, despite its significance, research on online hate speech has predominantly focused on subjects such as religion, race, political opinions, and women (Castaño-Pulgarín et al., 2021), with studies addressing hate expressions targeted at the elderly being relatively scarce.

Therefore, this study comprehends the trends of online gerontophobia and pinpoints its root causes, all with the objective of mitigating the escalating intergenerational conflicts in the digital sphere. In pursuit of this objective, the study seeks to define the concepts and types of gerontophobia. Additionally, it seeks to empirically examine the changing patterns of online gerontophobia within South Korean society by analyzing the news comment data on Naver, one of the most widely used online platforms in Korea. The specific research questions are as follows:

*RQ1*: How has the proportion of comments containing gerontophobic expressions changed over time in news discussions?

*RQ2*: Which types of gerontophobic expressions have seen an increase (or decrease)?

*RQ3*: If specific types of gerontophobia have increased, what are the underlying reasons?

To analyze the long-term patterns of gerontophobia, this study collected comments posted on news articles related to the elderly from May 1, 2017, to June 30, 2023. The collected comments were then processed using a classification model based on KcBert. This model was used to determine whether a comment exhibited gerontophobia and, if so, to label the specific type of gerontophobic comment.

The present study is structured as follows. In Section 2, a review of existing literature related to online hate speech is conducted to define the concept of gerontophobia and categorize its expressions. In Section 3, an overview of the entire research process is provided. Section 4 presents the analysis results, followed by the discussion and conclusion in Section 5 to conclude the study.

## 2 Literature review

### 2.1 Online hate speech

Hate speech refers to the act of demeaning specific groups based on factors such as gender, religion, race, disability, and more, stemming from prejudices (Jay, 2009). It goes beyond mere profanity or vulgar expressions; it involves insulting particular groups based on biases (Kim and Jung, 2021). European Commission against Racism and Intolerance (2016), opposing racial discrimination and indifference, defines hate speech as promoting hatred, humiliation, or underestimation in any form against individuals or groups, driven by motivations such as race, skin color, ancestry, nationality or ethnic origin, age, disability, language, religion or beliefs, gender, gender identity, sexual orientation, or other personal characteristics or conditions.

In the online realm, hate speech is characterized by using violent and aggressive language directed at specific groups through the internet and social networks. It is often motivated by ideologies or personal biases (Calderón et al., 2020). Particularly, the emotions of hatred and dislike inherent in prejudice tend to initially remain hidden within individuals and gradually become outwardly expressed and socially disseminated (Hong, 2019). As a result, online platforms, being unrestricted by physical constraints, facilitate the rapid spread of expressions of hatred, and furthermore, these expressions can persist for years. Online hate speech can persist over the years and rapidly spread beyond physical constraints, amplifying the impact of emotional contagion and leading to exacerbated negative consequences, including the internalization of negative values and the intensification of hatred (Kang, 2022).

Understanding and addressing online hate speech has been a longstanding endeavor. However, research specifically targeting hate speech targeting the elderly is notably deficient. According to Castaño-Pulgarín et al. (2021), an analysis of 67 articles on online hate speech or cyber-hate from 2015 to 2019 revealed that the studied target groups primarily included religion, race, political figures, and gender. While Meisner (2021) highlighted escalating age discrimination on social media following COVID-19, this too remained limited to trend commentary based on existing literature and lacked empirical analysis of the facets of online elderly hate. Within South Korea, some studies collected online comment data to analyze societal perceptions and attitudes toward the elderly (An et al., 2021), or examined public reactions to intergenerational conflicts (An et al., 2022). However, the quantity and diversity of such research still fall short.

Within South Korea, generational conflict and hatred toward the elderly are particularly pressing issues. Furthermore, 2020 witnessed the global outbreak of COVID-19, highlighting the vulnerability of the elderly population, especially in the United States. This period also saw the emergence of a term “Boomer Remover,’ which, in a somewhat derogatory sense, referred to the idea that the disease was removing the older generation. Such intergenerational hate and conflicts are not limited to Korea but have been observed in various parts of the world. Hence, there is a need for a range of studies aimed at enhancing the understanding of online hate directed at the elderly. Accordingly, our study aims to longitudinally analyze the nature of online hate directed at the elderly within the context of South Korea, contributing to the mitigation of generational conflicts and filling the existing academic gaps.

Online hate speech has been categorized into various types in previous studies. Miró Llinares (2016) addressed the issue of certain expressions or forms of violent communication in cyberspace amplifying negative tones, analyzing around 250,000 Spanish tweets. They classified online violent communication and hate speech into three types based on Twitter data: “Directly promoting or praising violence”, “Encouraging discrimination, hate, or restricting rights”, and “Insulting to emotion”.

Reichelmann et al. (2021) investigated cross-national similarities and differences in online hate speech content, exposure, and emotional reactions. Conducting online surveys among 18 to 25-year-olds from six countries (Finland, France, Poland, Spain, United Kingdom, and United States), they found that the majority of respondents unintentionally encountered online hate within the preceding three months. Their survey included the question, “What type of hate have you encountered?” and they categorized hate speech into six types: “Advocate Violence against Group”, “Use Stereotypes to Describe Group”, “Blame Group for Personal Problems”, “Blame Group for National Problems”, “Advocate Hatred of Group”, and “Call for Discrimination against Group”. Survey results revealed that respondents were primarily exposed to the “Use Stereotypes to Describe Group”, “Advocate Hatred of Group”, and “Call for Discrimination against Group” types.

Yang (2022) focused on the surge of hate comments on online news portals, analyzing crime news articles from Naver, a prominent platform in Korea. They identified and analyzed hate comments targeting women, immigrants, and the elderly. Prior to analysis, they categorized hate types into six categories: “Labeling”, “Stereotyping”, “Separating”, “Simple Nominating”, “Mocking”, and “Other”. According to this study, hate directed toward the elderly, extracted from crime news articles, primarily involved stereotyping the elderly as politically extreme right-wing conservatives and socially separating them.

### 2.2 Characteristics of online gerontophobia

Elderly prejudice and discrimination have been broadly defined as negative feelings and actions directed toward older individuals or groups representing them within society (Butler, 1989; Ayalon and Tesch-Römer, 2017; Lee, 2017; Hong, 2019; Shin and Choi, 2020; Lee and Song, 2021; Kang, 2022). In this study, drawing upon the prior research discussed in Section 2.3, online gerontophobia is defined as “expressions of criticism, disdain, or insult rooted in bias toward the elderly group occurring in online and social network environments.”

According to previous research, elderly prejudice exhibits various characteristics and manifestations: Firstly, elderly prejudice reflects negative fears and anxieties toward older individuals, often stemming from the perception of older people as economically or socially vulnerable (Lee and Song, 2021; Kang, 2022). Lee (2017) discussed it as aversion toward individuals perceived as threats to one’s survival or reproduction, such as economically disadvantaged or socially marginalized individuals. Older adults are particularly prone to becoming targets of this kind of prejudice, as they are often seen as socially unproductive and of little value, inducing fear among other members of society (Butler, 1989; Lee and Song, 2021). Secondly, elderly prejudice includes perceptions that exaggerate the physical and psychological characteristics of older individuals or evaluate them as antisocial groups incapable of adapting to societal norms (Ayalon and Tesch-Römer, 2017; Hong, 2019; Shin and Choi, 2020; Lee and Song, 2021). Thirdly, elderly prejudice is accompanied by an instinctive aversion and discomfort, driven by the desire to distance oneself from it (Rozin et al., 1999; Lee and Song, 2021; Kang, 2022). In society, older individuals are seen as a minority with different physical characteristics compared to the majority of younger generations. At this point, the perception of older adults as an alien presence can trigger an instinctive aversion, often stemming from an unwillingness to acknowledge negative aspects within oneself (Kang, 2022). Fourthly, elderly prejudice can also be attributed to limited understanding due to a lack of information (Lee and Song, 2021). This limited comprehension of the factors and processes associated with aging and viewing older adults from a restricted viewpoint contributes to elderly prejudice. Finally, elderly prejudice is expressed in linguistic forms and behaviors that discriminate against and exclude older individuals across various domains (Hong, 2019; Shin and Choi, 2020; Lee and Song, 2021; Yang, 2022). This includes expressions and actions that criticize or demean older individuals, potentially leading to their social isolation and the restriction of their rights.

In this study, we have integrated the characteristics of online hate and gerontophobia as defined in prior research to categorize online gerontophobia into the following five types, reflecting its causes: (1) Fear of Aging, (2) Resource Burden, (3) Social Isolation, (4) Criticism of Social Behavior, and (5) Stereotypes of Political Orientations. “Fear of Aging” refers to an innate aversion toward aging itself, including derogatory attitudes toward the physical characteristics of older individuals (e.g., appearance, ailments) and the negative use of terms related to aging. “Resource Burden” can be defined as prejudice expressions that perceive older individuals as competitors threatening one’s own survival and the allocation of social and economic resources. “Social Isolation” signifies prejudice that views older individuals as non-productive entities detrimental to societal progress, considering them as no longer necessary in society. This may involve the exclusionary perception of older individuals and expressions that advocate or justify restrictions on their rights. “Criticism of Social Behavior” denotes prejudice that assumes older individuals naturally possess deviant behavior and are incapable of social adaptation, perceiving them as socially maladjusted entities. This may encompass criticism of inappropriate social behavior and manners exhibited by older individuals. Lastly, “Stereotypes of Political Orientations” refers to prejudice based on stereotypes that older individuals inherently hold extremely conservative political views. This includes using derogatory expressions toward older individuals in criticism of specific political parties’ policies or political groups and criticizing older individuals’ political behavior (Tables 1, 2).

**Table 1:** Types of gerontophobia.

Type of Gerontophobia	Description
Fear of aging	Innate aversion toward different beings (e.g., appearance-based derogatory comments, odor, etc.) Contempt for the physical attributes Unwarranted insults, offensive language, and mockery
Social burden	Perceived as a threat in terms of one’s own survival and resource allocation
Social isolation	A hindrance to innovation, unproductive, and an impediment to social progress Considered as undervalued, unproductive, and unnecessary existence
Criticism of social behavior	Shifting blame onto the elderly, promoting and fostering hatred and violence Stereotypes about social manners and attitudes
Stereotypes about political orientations	Stereotypes regarding political inclinations.

**Table 2:** Previous research on online hate and gerontophobia.

	Author	Concepts or types of hatred	Type of Gerontophobia
Online Hate	Miró (2016)	Directly promoting or praising violence	3, 4
		Encouraging discrimination, hate, or restricting rights	3, 4
		Insulting to emotions	3
	Calderón et al. (2020)	Making false or irrelevant accusations	4
		Promoting, advocating, justifying, or praising physical violence against groups	3, 4
		Using offensive messages or derogatory terms against group’s emotions or beliefs	3, 5
		Demanding or justifying restrictions on group’s rights	3
	Reichelmann et al. (2021)	Advocating violence against groups	3, 4
		Using stereotypes to describe groups	1, 2, 3, 4, 5
		Diverting personal issues onto groups	4
		Assigning national issues to groups	4
		Endorsing hate toward groups	3, 4
		Demanding discrimination against groups	3, 4
	Yang (2022)	Labeling: assigning unique names to target groups	1, 2, 3, 4, 5
		Stereotyping: mentioning negative fixed perceptions and prejudices about the target group	1, 2, 3, 4, 5
		Separating: treating the target group as something distinct	3
		Simple nominating: simple naming of the target group with expressed hate	1, 2, 3, 4, 5
		Mocking: using sarcasm, ridicule, or irony to mock the target group	1, 2, 3, 4, 5
		Other: cases not fitting in specific categories but containing hate nuances	–
Gerontopobia	Ayalon and Tesch-Römer (2017)	Prejudice, stereotypes, and beliefs about age	1, 2, 3, 4, 5
	Hong (2019)	Promote negative stereotypes or biases	4, 5
		Scorn, insult, or threaten	1, 4, 5
		Incite discrimination or hatred	3, 4
	Shin and Choi (2020)	Containing slander and disregard	1, 4, 5
		Considering as a presence that should disappear	3
		Rejecting as a selfish and antisocial alien group	3, 4
	Lee and Song (2021)	Targets perceived as threats to one’s survival or reproduction	2
		Non-productive and worthless existence	2, 3
		Presence with illnesses	1
		Engaging in exaggerated behaviors to elicit reactions or attention	4
		Innate aversion	1
		Verbal violence (attitudes that exert inappropriate force through words, tone, nonverbal cues, etc.)	1
		Discriminatory acts and defamation that ignore the experiences and needs of the elderly	3
		Group pushed outward while productivity and creativity are ignored	2, 3
	Kang (2022)	Innately felt hatred	1
		Contemptible and vile targets of hatred	1, 4
		Othering based on differences	3

* (1) Fear of Aging, (2) Resource Burden, (3) Social Isolation, (4) Criticism of Social Behavior, (5) Stereotypes of Political Orientation.

## 3 Data and methods

**Figure 1:**
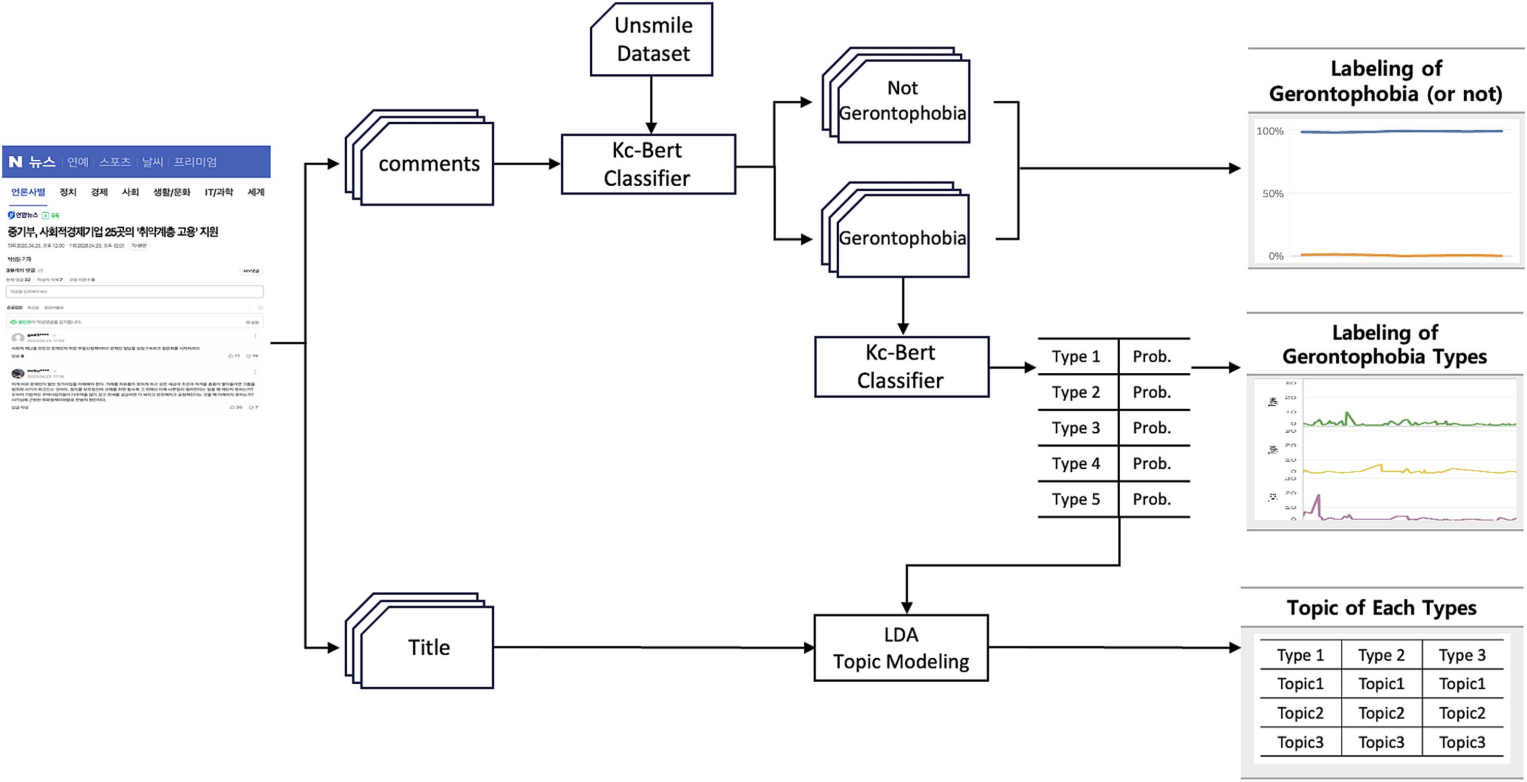
Overview of the analysis process.

This study aims to understand the shifting patterns of gerontophobia in the online realm and to delve into the fundamental causes of gerontophobia. To achieve this, we conducted an analysis that involved (1) collecting article information and comment data, (2) labeling for the presence and types of gerontophobia, and (3) conducting LDA Topic Modeling to the title of articles based on the types of gerontophobia. The overall analytical process is illustrated in Figure 1.

### 3.1 Data collection

To collect gerontophobic comments, Naver News was chosen as the platform. As of 2023, Naver is a prominent search engine in South Korea, holding an average market share of 58.18% among Korean search engines (Internet Trend, 2023). Particularly, Naver News serves as a platform that collects articles from over 70 media outlets and provides news aggregation services. It has been extensively utilized in research to analyze online opinions and commenting behaviors (Kwak et al., 2018; Baek et al., 2020, 2022; Lee and Lee, 2023).

Today, the issues of population aging and generational conflict in South Korean society can be categorized into the domains of politics, economy, and social culture (Hwang, 2022). Based on this categorization, we collected information on articles related to the elderly within Naver News under the categories of politics, economy, and society. This information included news IDs, publication dates, and news URLs. Additionally, we gathered data on comments related to these articles, including commenter IDs, comment timestamps, and comment content. At this point, we assessed whether the article titles contained keywords related to the elderly as a criterion to determine if they contained topics related to the elderly.

For data collection, we accessed the Naver News website using Python’s “requests’ and “BeautifulSoup’ modules to scrape information from articles and comments. And to analyze the long-term patterns of elderly prejudice, data collection was carried out from May 1, 2017, to June 30, 2023. During this period, a total of 133,218 articles related to the elderly were uploaded, and 1,238,935 comments were collected. After removing duplicate comments, missing values, and comments with fewer than five characters, we proceeded with the analysis using a total of 760,140 comments. Information on the data collection results for each category of politics, economy, and society is presented in Table 3.

**Table 3:** Information on the collected comments.

Type	The number of collected articles	The number of collected comments	The number of comments after preprocessing
Politics	12,663	227,108	167,700
Economy	21,679	120,330	83,977
Society	98,876	891,497	516,573
Total	133,218	1,238,935	760,140

### 3.2 Labeling of gerontophobia comments

Deep learning models such as attention-based multi-channel CNN (Lee and Lee, 2023), LSTM (Kim J. et al., 2020), KoBERT & KoGPT2 & KoELECTRA (Lee and Park, 2022), and CNN-LAN (Park et al., 2023) are being utilized for hate speech detection in Korean language data. In this study, to label the presence of elderly prejudice and the types of elderly prejudice in the comments, we utilized the KcBERT classifier, which is based on BERT (Bidirectional Encoder Representations from Transformers). BERT is a language model released by Google, trained on English text data, known for its ability to capture contextual information in text, and it is recognized as one of the top-performing deep learning-based language models (Geetha and Karthika Renuka, 2021; Prottasha et al., 2022). Google has also released mBERT, a multilingual BERT model trained in 104 languages in addition to English. BERT, being a bidirectional language model, allows researchers to fine-tune pre-trained models not only for binary classification tasks but also for multiclass classification problems, making it a versatile tool for various research purposes (Arslan et al., 2021; Geetha and Karthika Renuka, 2021; Li et al., 2022; Qasim et al., 2022).

Various models, including KoBERT, KorBERT, and KcBERT, have been publicly released, trained specifically for Korean using only Korean data. These models aim to optimize BERT for the Korean language (Lee et al., 2021). Among these, KcBert collected approximately 110 million Naver News comments from January 2019 to June 2020. After minimal preprocessing, it was trained with the Huggingface BERT WordPiece tokenizer, making it one of the most widely used models in commercial applications (Jang et al., 2022). For this reason, KcBERT can be considered the most suitable Korean Bert model for our target dataset, Naver News comments.

#### 3.2.1 Labeling whether a comment contains gerontophobia or not

It is necessary to determine whether the collected comments are expressions of elderly prejudice or not and label them accordingly. For this purpose, in this study, we utilized the Korean Hate Speech dataset “UnSmile” provided by Smilegate AI. This dataset defines hate speech as expressions that reproduce hostile remarks, mockery, caricatures, and prejudices against specific social (minority) groups and tags them into various categories, including women/family, men, sexual minorities, race/nationality, age, region, religion, other forms of hate, abusive language, and clean. Table 3 provides an overview of the “UnSmile” dataset. To enhance the classification performance for elderly prejudice, we incorporated 684 comments related to gerontophobia from our collected comment dataset, labeling them under the “age” category for training the model. Out of the entire dataset, 80% was used as training data and 20% as test data for classifier training. The trained classifier exhibited respectable gerontophobic expression classification performance with a precision of 0.98, recall of 0.78, and an F1-score of 0.87 (Table 4). The classification performance for each label is presented in Table 5. Using the trained model, we automatically classified whether the remaining 759,456 comments, excluding the 684 comments used in the training, contained gerontophobic express or not. As a result, 5,643 comments were initially classified as gerontophobia comments.

**Table 4:** Information on the Unsmile dataset.

	Women /Family	Men	Sexual minorities	Race/Nationality	Age (incorporated)	Region	Religion	Other forms of hate	Abusive language	Clean
The number of sentences	1,399	1,681	1,418	2,154	749 (1,433)	1,312	1,471	703	3,932	4,674

* Abusive Language: a sentence that includes derogatory or insulting remarks about someone’s appearance or others without specifying the targeted group, causing discomfort, and containing offensive or explicit content.

* Clean: a neutral sentence that does not contain offensive language, profanity, discomfort, or explicit content.

**Table 5:** The classification performance for each target label.

	Precision	Recall	F1-score
Women/Family	0.79	0.80	0.79
Men	0.84	0.84	0.84
Sexual Minorities	0.85	0.86	0.85
Race/Nationality	0.84	0.80	0.82
Age	0.98	0.78	0.87
Region	0.84	0.87	0.85
Religion	0.87	0.90	0.88
Other Forms of Hate	0.71	0.13	0.22
Abusive Language	0.70	0.62	0.66
Clean	0.76	0.62	0.68

#### 3.2.2 Labeling comments containing gerontophobia by gerontophobia type

To construct a dataset for training a model to classify types of ageist expressions, we randomly sampled 1,920 comments from the 5,643 comments classified as ageist. Two researchers, following the defined definitions of gerontophobia types, jointly discussed and labeled each comment with its corresponding type. However, since a deep learning-based classifier was utilized here, there is a possibility of non-gerontophobic comments being included. Therefore, to construct a more accurate dataset for classifying types of gerontophobia, separate labels were assigned to comments that did not contain expressions of gerontophobia. The labeling results are shown in Table 5. Of this dataset, 80% was used for training, and 20% for testing the classifier. The classifier’s performance, categorized by labels, is presented in Table 6. Using the trained model, automatic labeling of gerontophobia types was performed on 3,723 comments out of the 5,643 comments classified as gerontophobia, excluding the 1,920 comments used for training. As a result, the final classification yielded 757,886 comments (99.70%) classified as non-gerontophobic comments and 2,254 comments (0.30%) as gerontophobic comments. Among the five gerontophobia types, 348 were classified as “Fear of Aging,” 632 as “Resource Burden,” 538 as “Social Isolation,” 133 as “Criticism of Social Behavior,” and 203 as “Stereotypes of Political Orientations.”

**Table 6:** Information on the type of gerontophobic expression classification dataset.

	Non-Gerontophobia	Fear of aging	Resource burden	Social isolation	Criticism of social behavior	Stereotypes of political orientations
The number of comments	953	173	225	233	133	203

### 3.3 LDA topic modeling

At this step, we aimed to investigate the underlying causes of each type of gerontopobic expression. Since the societal events or topics triggering these expressions for each type may be similar or different, the direction to mitigate gerontophobia for each type can vary depending on these causes. For this purpose, LDA topic modeling was utilized to extract relevant topics from news article titles for the top 20 dates with the highest occurrences of each previously categorized type of gerontophobia. Information regarding the top 20 days for the appearance of gerontophobic expressions for each type is provided in Table 5, as shown below.

Topic modeling is a widely used technique in natural language processing, aiming to discover the underlying meaning conveyed by words in documents and extract topics from them (Maier et al., 2018; Jelodar et al., 2019). Among these techniques, Latent Dirichlet Allocation (LDA) clusters documents based on the probability and distribution of keywords, inferring topics, and estimating the likelihood of keywords being included in each topic (Maier et al., 2018). As a result, LDA can not only infer the primary topics and keywords within documents but also reverse-engineer the most relevant topics for each document. The topics derived from LDA represent the core themes embedded in the documents, and the keywords associated with each topic serve as representative terms for those topics. LDA is known for its relative simplicity in extracting information about hidden topics from large-scale documents and its high utility and scalability (Blei et al., 2003), making it widely applied in various natural language processing studies (Maier et al., 2018; Gupta et al., 2022).

A critical aspect of LDA topic modeling lies in determining the number of topics. Due to the subjective nature of topic modeling, the researcher’s judgment inevitably plays a role in determining the number of topics. Therefore, to minimize researcher bias, Perplexity and Coherence were consulted. Perplexity is a measure of how well the estimated model can generate a given set of documents, and Coherence measures the semantic similarity of the top-weighted words within each topic (Kirilenko et al., 2021). To do this, LDA Gibbs modeling was executed 1,000 times for news article title data for each gerontophobia type. Based on the results, the number of topics for each gerontophobia type was determined as follows: “Fear of Aging” with 9 topics, “Resource Burden” with 6 topics, Social Isolation with 6 topics, “Criticism of Social Behavior” with 4 topics, and “Stereotypes of Political Orientations” with 7 topics.

## 4 Result

### 4.1 Trends of online gerontophobia expressions

**Figure 2:**
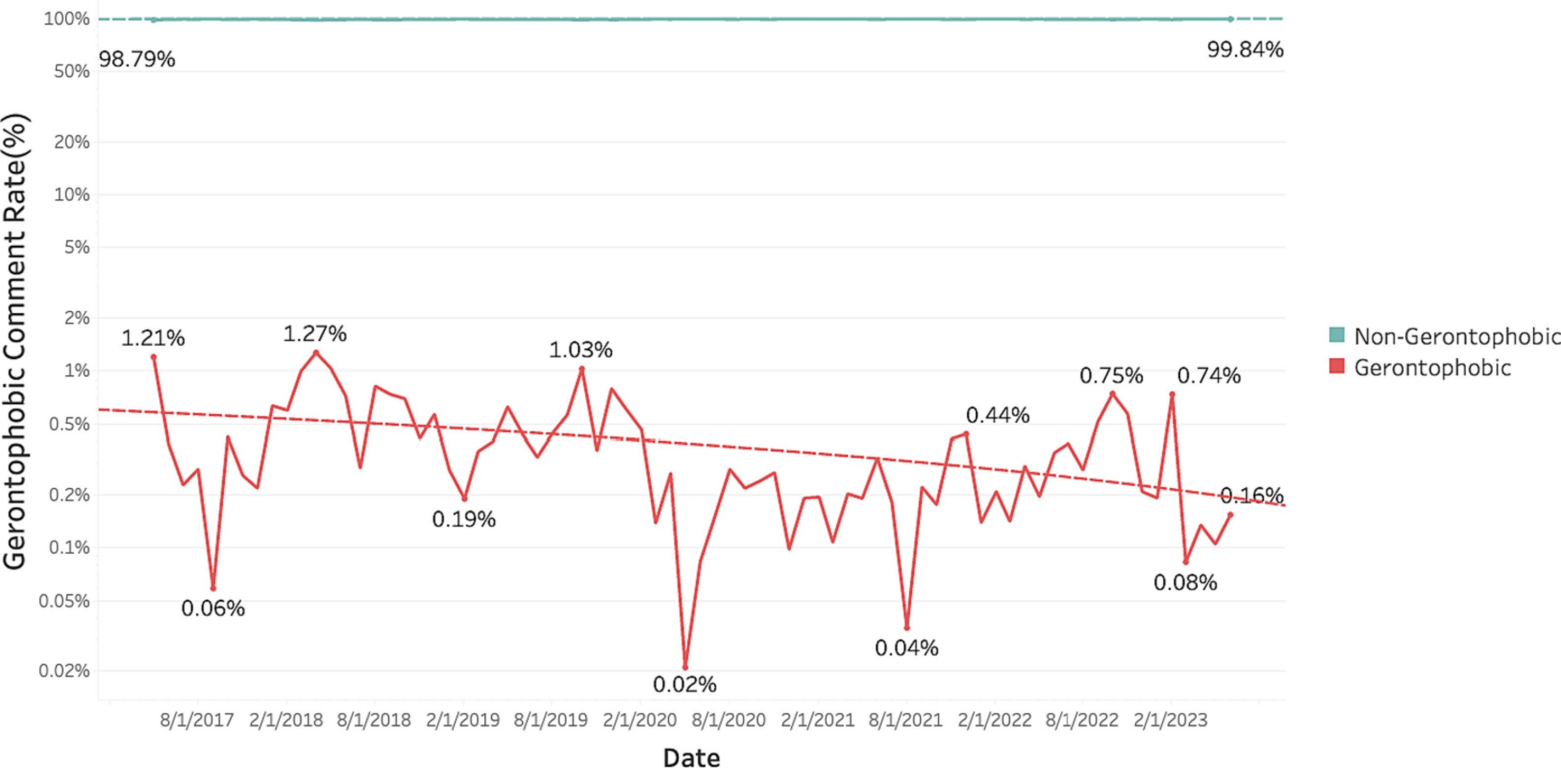
Monthly changes in the ratio of gerontophobic comments.

To address the first research question, that “How has the proportion of comments containing gerontophobic expressions changed over time in news discussions?,” we visualized the changes in the proportion of comments containing gerontophobic expressions over time using KcBERT classification results. Figure 2 presents a graphical representation of the monthly evolution, from May 2017 to June 2023, of the ratio of gerontophobic comments among all comments, displayed on a logarithmic scale. According to our classification results, except for months where there were no gerontophobic comments at all, the highest proportion of gerontophobic comments within the total comments was 1.21%, while the lowest proportion was only 0.02%. Furthermore, when observing the trend, we confirmed a gradual but consistent decrease in the proportion of gerontophobic comments over a period of approximately six years, as detailed in Table 6.

**Figure 3:**
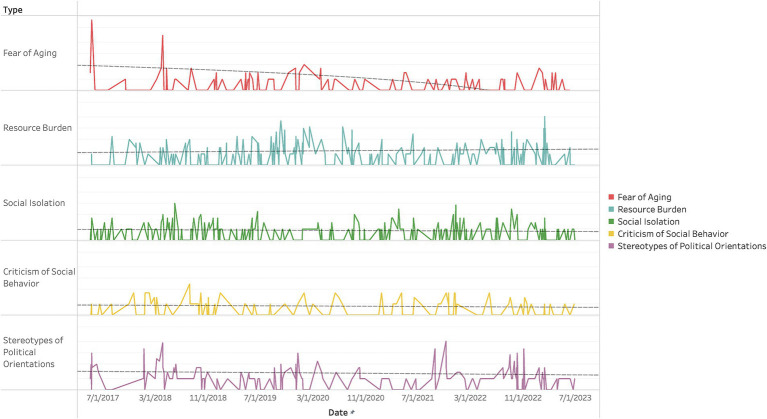
Daily changes in the gerontophobic comments of each type.

To address the second research question, “Which types of gerontophobic expressions have seen an increase (or decrease)?,” we conducted an analysis based on the classification of 2,254 comments identified as gerontophobic into five categories. We visualized and performed trend analysis on the patterns of gerontophobic expressions from May 2017 to June 2023, and the results are presented in Figure 3 and Table 7.

**Table 7:** The classification performance for gerontophobia type label.

	Precision	Recall	F1-score
Non-gerontophobia	0.46	0.73	0.56
Fear of aging	0.88	0.23	0.37
Resource burden	0.64	0.51	0.57
Social isolation	0.65	0.29	0.40
Criticism of social behavior	0.96	0.40	0.56
Stereotypes of political orientation	0.79	0.66	0.72

Our analysis revealed that expressions falling under the category of “Fear of Aging” exhibited spikes in occurrence on specific dates. Furthermore, the trend analysis showed a gradual but statistically significant decrease in these expressions from May 2017 to June 2023. In contrast, the categories “Resource Burden,” “Social Isolation,” and “Criticism of Social Behavior” did not display significant changes in the graphical representation, and the trend analysis also indicated no substantial variations in their frequency over the same time period. Lastly, the category “Stereotypes of Political Orientations” exhibited occasional spikes in comment frequency like the “Fear of Aging” category on specific dates. However, no statistically significant differences were observed when considering the overall trend from May 2017 to June 2023.

### 4.2 Key issues in each gerontophobia type

Based on the results of topic modeling, the major topics in the news for each type of gerontophobia are consistent with those presented in Table 8. Specifically, under the category of “Fear of Aging,” significant topics included Comfort Women Issue (Historical Event), COVID-19 Confirmed Cases, Digital & Financial Exclusion, Elderly Employment & Labor, Elderly Poverty, Elderly Welfare (Pension, etc.), Health Care, National Holidays (Seniors’ Day, etc.), and Politicians’ Actions. For the “Resource Burden” category, the primary topics were Digital & Financial Exclusion, National Fiscal Deterioration, Elderly Employment & Labor, Elderly Crime & Accidents, Elderly Welfare (Pension, etc.), and Politicians’ Actions. In the case of “Social Isolation,” the prominent topics comprised COVID-19 Vaccination, Elderly Crime & Accidents, Elderly Welfare (Pension, etc.), Health Care, Politicians’ Actions, and Voting. Within the “Criticism of Social Behavior” type, major topics included COVID-19 Confirmed Cases, Elderly Crime & Accidents, and Elderly Welfare (Pension, etc.). Lastly, for the “Stereotypes of Political Orientations” category, the main topics encompassed Comfort Women Issue (Historical Event), Digital & Financial Exclusion, Elderly Crime & Accidents, Elderly Poverty, Elderly Welfare (Pension, etc.), Politicians’ Actions, and Voting.

**Table 8:** Top 20 days with the appearance of gerontophobic comment for each type.

Fear of aging		Resource burden		Social isolation		Criticism of social behavior		Stereotypes of political orientations	
Date	# of comment	Date	# of comment	Data	Count	Date	# of comment	Date	# of comment
05.08.2017	81	02.07.2023	21	05.28.2018	10	08.05.2018	7	11.06.2021	21
04.02.2018	31	10.02.2019	16	12.22.2021	9	11.18.2017	4	04.02.2018	19
01.18.2020	5	02.12.2020	11	04.01.2021	7	01.10.2018	4	01.05.2018	13
03.26.2018	4	07.15.2020	11	09.06.2022	7	01.30.2018	4	09.16.2021	13
04.20.2018	4	01.15.2020	10	06.16.2019	6	09.26.2018	4	11.02.2022	13
08.12.2018	4	02.03.2023	10	04.03.2018	5	12.25.2018	4	05.08.2017	10
12.09.2019	4	02.09.2023	10	09.26.2018	5	01.18.2020	4	12.20.2019	10
04.04.2020	4	10.20.2019	9	09.07.2020	5	06.10.2020	4	10.02.2022	10
01.12.2023	4	08.27.2020	9	12.10.2021	5	01.11.2021	4	08.30.2022	9
05.01.2017	3	09.11.2019	8	10.02.2022	5	04.14.2021	4	03.05.2018	7
12.07.2018	3	09.06.2022	8	05.08.2017	4	11.07.2021	4	03.18.2018	6
04.22.2019	3	09.27.2019	7	04.02.2018	4	11.18.2021	4	03.20.2020	6
05.10.2019	3	06.06.2021	7	04.24.2018	4	12.15.2021	4	09.19.2022	6
06.18.2019	3	08.10.2017	6	05.18.2018	4	12.18.2021	4	05.05.2017	5
11.07.2019	3	03.13.2019	6	08.24.2018	4	05.26.2022	4	05.31.2017	5
12.27.2019	3	05.21.2019	6	09.18.2018	4	02.22.2018	3	06.02.2018	5
04.26.2021	3	08.18.2019	6	11.22.2018	4	03.05.2018	3	10.02.2019	5
05.05.2021	3	12.11.2019	6	12.13.2018	4	10.31.2018	3	12.11.2019	5
02.07.2022	3	10.29.2017	5	05.22.2019	4	06.27.2020	3	08.31.2022	5
10.02.2022	3	04.19.2018	5	02.27.2021	4	06.12.2021	3	10.04.2022	5

A wide array of concerns and issues related to various types of gerontophobia are prominently featured. Firstly, the presence of COVID-19-related topics across multiple types of gerontophobia signifies the significant impact of the pandemic on perceptions and discussions regarding the elderly. It underscores the need for comprehensive measures that consider societal awareness when protecting and supporting the elderly during national crises threatening health, such as disease outbreaks.

Furthermore, the recurring themes of digital and financial exclusion indicate their critical relevance to gerontophobia. Particularly, the presence of gerontophobic expressions stemming from themes related to social isolation among the elderly suggests an urgent necessity for improving the perception of elderly individuals as integral members of society.

Of noteworthy concern is the emergence of economic and social welfare-related topics across all types of gerontophobia. Topics such as elderly employment, job opportunities for seniors, elderly poverty, and pensions are ultimately linked to national fiscal challenges. This leads to perceptions of the elderly as resource-draining entities, dispensable to society, and even isolated to the extent of being denied fundamental rights, including voting.

Moreover, the frequent utilization of gerontophobic expressions concerning politicians’ actions implies that negative terms associated with the elderly and aging are prevalent. This reflects a negative societal perception of the elderly and aging itself.

Lastly, the association of gerontophobia with historical events like the comfort women issue underscores the importance of understanding historical contexts. It emphasizes that bridging the generation gap and fostering an understanding of historical events are essential steps in addressing gerontophobia (Tables 9–11).

**Table 9:** Information of the trendline model for monthly trends in gerontophobic comments ratio.

	*p*-value (Line)	Label	Parameter estimate	Standard error	*t*-value	*p*-value
Gerontophobic	0.000	Intercept	0.081	0.020	3.9641	0.000
		Month	−1.746e-06	4.627e-07	−3.774	0.000

**Table 10:** Information of the trendline model for daily trends in gerontophobic comments of each type.

	*p*-value (Line)	Label	Parameter estimate	Standard error	*t*-value	*p*-value
Fear of aging	0.025*	Intercept	88.800	38.277	2.320	0.022*
		Date	19.641e-04	8.691e-04	−2.260	0.025*
Resource burden	0.363	Intercept	−7.123	10.485	−0.679	0.498
		Date	2.169e-04	0.000	0.911	0.363
Social isolation	0.362	Intercept	6.185	4.781	1.294	0.197
		Date	−9.925e-05	1.086e-04	−0.914	0.362
Criticism of social behavior	0.424	Intercept	6.288	5.685	1.106	0.271
		Date	1.037e-04	1.293e-04	−0.802	0.424
Stereotypes of political orientations	0.391	Intercept	14.815	13.987	1.060	0.291
		Date	−2.726e-04	3.173e-04	−0.859	0.391

**Table 11:** Topics in news by type of gerontophobia.

Fear of aging	Resource burden	Social isolation	Criticism of social behavior	Stereotypes of political orientations
Comfort Women Issue (Historical Event) COVID-19 Confirmed Cases Digital and Financial Exclusion Elderly Employment and Labor Elderly Poverty Elderly Welfare (Pension, etc.) Health Care National Holidays (Seniors’ Day, etc.) Politicians’ Actions	Digital and Financial Exclusion National Fiscal Deterioration Elderly Employment and Labor Elderly Crimes and Accidents Elderly Welfare (Pension, etc.) Politicians’ Actions	COVID-19 Vaccination Elderly Crimes and Accidents Elderly Welfare (Pension, etc.) Health Care Politicians’ Actions Voting	COVID-19 Confirmed Cases Elderly Crimes and Accidents Elderly Welfare (Pension, etc.) Health Care	Comfort Women Issus(Historical Event) Digital and Financial Exclusion Elderly Crimes and Accidents Elderly Poverty Elderly Welfare (Pension, etc.) Politicians’ Actions Voting

* The order of the topics is simply arranged in ascending order and is not related to the importance of the topics.

## 5 Discussion

In this study, we focused on the societal issue of South Korea’s rapidly aging population and the resulting intensification of generational conflicts. Our objective was to explore the current status and underlying causes of gerontophobia in online contexts. To achieve this, we initially categorized gerontophobia into five types: “Fear of Aging,” “Resource Burden,” “Social Isolation,” “Criticism of Social Behavior,” and “Stereotypes of Political Orientation,” based on a literature review that provided insights into the causes of gerontophobia.

Furthermore, for empirical analysis of the changing patterns of gerontophobia, we collected and analyzed news comments from Naver, the most widely used news platform in South Korea. To capture long-term trends, we collected information on elderly-related news articles spanning approximately six years, from May 1, 2017, to June 31, 2021, and analyzed a total of 760,140 comments. To classify whether comments contained expressions of gerontophobia and, if so, what type of gerontophobic expressions were present, we trained and utilized a kc-BERT-based classifier.

The results indicated that out of 760,140 comments, only 2,254 contained expressions of gerontophobia, accounting for 0.30% of the total comments. This suggested that gerontophobia was not prevalent at a severe level in the examined context. Additionally, when examining the overall trend of gerontophobia expressions from May 2017 to June 2023, it appeared that expressions of gerontophobia had, in fact, decreased over time. Analyzing the changing trends of gerontophobic expressions for each predefined type, we observed a significant decrease in the “Fear of Aging” category, while the other types showed no significant changes. Taken together, the overall decrease in the prevalence of gerontophobic expressions appeared to be mainly influenced by the reduction in expressions related to the “Fear of Aging” type.

In light of these considerations, there is a need to reevaluate the discussion on the increasing gerontophobia and intergenerational conflicts in traditional media and press. According to the 2021 “Online Hate Speech Awareness Survey” conducted by the National Human Rights Commission of Korea, the primary cause of hate speech expressions was identified as “the attitude of the media (79.2%),” and 76.3% of respondents stated that they no longer considered it a problem when politicians and public figures used hate speech expressions (National Human Rights Commission of Korea, 2021). When we combine the results of this survey with the findings of our study, it suggests the possibility that the extent of hatred experienced by people due to the social influence of politicians and media may differ from what is depicted in the media.

To further understand the factors contributing to the increase or decrease in each type of gerontophobic expression, additional analysis was conducted using LDA Topic Modeling on the top 20 news article titles for each type that was most frequently captured. The results of the topic modeling revealed a total of 9 topics for “Fear of Aging,” 6 topics for “Resource Burden,” 6 topics for “Social Isolation,” 4 topics for “Criticism of Social Behavior,” and 7 topics for “Stereotypes of Political Orientations.”

When considering the comprehensive results, we have discovered that when discussing generational conflicts, it is essential to consider various aspects of the factors that generate ageist expressions. This implies the need to consider various aspects of the elderly population, including elderly welfare, elderly poverty, elderly digital and social exclusion, and elderly healthcare. It also suggests the importance of recognizing persistent challenges resulting from unresolved domestic historical events such as the “Comfort Women Issue” and national disasters like COVID-19.

## 6 Conclusion

In light of these factors, there is a strong emphasis on the need for a collective effort to reduce gerontophobia. This effort should involve various stakeholders, including policymakers, to foster positive perceptions of the elderly population and establish supportive systems for their well-being.

The academic significance of this study lies in its attention to online gerontophobia and its categorization of the causes of gerontophobia based on existing research. This establishes a foundation for future research on gerontophobia. Furthermore, unlike other studies that primarily utilize machine learning methodologies, this research aimed to enhance classification performance by using KcBERT.

From a practical standpoint, this research contributes to data-driven decision-making and the mitigation of intergenerational conflicts. It provides foundational data for a more detailed consideration of intergenerational conflicts and offers a process to monitor changes in gerontophobia triggered by specific events. This, in turn, allows for the management of online gerontophobia through policy adjustments based on policy level and timing.

However, there are certain limitations to this study. Firstly, it focused on analyzing hate speech directed toward a specific target group, the elderly, which may have limited data availability. To train a classifier for the five types of ageist expressions, a sufficient amount of data is essential. However, in this study, we only used 1,920 comment data for the type of classification training phase, with each type consisting of approximately 100 to 200 comments. The somewhat less overwhelming performance presented in Table 6 is attributed to these limitations in data quantity. Therefore, future efforts should focus on obtaining more ageist comments to address these constraints. Additionally, in this study, we concentrated on analyzing comment data collected from the Naver News platform exclusively. However, in the extension of this study, considering the growing role of social media platforms like YouTube in information dissemination, it may be worthwhile to attempt data collection from a broader range of platforms. Secondly, the interface of Naver’s news and comment functions has undergone significant changes from 2017, the period of this study, to 2023. While these changes may have had an impact on the overall comment activity of users, this aspect was not considered in our study. Therefore, future research should attempt trend analysis while considering these environmental factors. Lastly, an in-depth examination of events and causes that influence the patterns of ageist expressions will be a valuable avenue for future research. This could involve reviewing whether these events align with societal, cultural, political, and historical events during the study period.

## Data availability statement

The raw data supporting the conclusions of this article will be made available by the authors, without undue reservation.

## Author contributions

SK: Data curation, Writing – original draft, Methodology. MR: Data curation, Writing – original draft, Conceptualization.
